# Factors associated with early, late, and very late stent thrombosis among patients with acute coronary syndrome undergoing coronary stent placement: analysis from the ATLAS ACS 2-TIMI 51 trial

**DOI:** 10.3389/fcvm.2023.1269011

**Published:** 2024-01-08

**Authors:** Gerald Chi, Fahad AlKhalfan, Jane J. Lee, Sahar Memar Montazerin, Clara Fitzgerald, Serge Korjian, Wally Omar, Elliot Barnathan, Alexei Plotnikov, C. Michael Gibson

**Affiliations:** ^1^ Division of Cardiovascular Medicine, Department of Medicine, Beth Israel Deaconess Medical Center and Harvard Medical School, Boston, MA, United States; ^2^ Department of Trial Design and Development, Baim Institute for Clinical Research, Boston, MA, United States; ^3^ Cardiovascular, Metabolism, Retina and Pulmonary Hypertension, Johnson & Johnson Pharmaceutical Research and Development, Raritan, NJ, United States

**Keywords:** stent thrombosis (ST), acute coronary syndrome (ACS), percutaneous coronary intervention (PCI), myocardial infarction (MI), rivaroxaban, factor Xa inhibitor, anticoagulant, landmark analysis

## Abstract

**Background:**

Stent thrombosis (ST) is an uncommon but serious complication of stent implantation. This study aimed to explore factors associated with early, late, and very late ST to help guide risk assessment and clinical decision-making on ST.

**Methods:**

The analysis included patients who received stent placement for the index acute coronary syndrome (ACS). Cumulative incidence of ST was assessed at 30 days (early ST), 31–360 days (late ST), 361–720 days (very late ST), and up to 720 days. Cox proportional hazards models were used to assess associations between ST and various factors, including patient characteristics [i.e., age, sex, ACS presentation, history of hypertension, smoking, diabetes, prior myocardial infarction (MI), heart failure, prior ischemic stroke, and cancer], laboratory tests [i.e., positive cardiac biomarker, hemoglobin, platelet count, white blood cell (WBC) count], and treatment [i.e., drug-eluting stent (DES) vs. bare-metal stent (BMS) and anticoagulant with rivaroxaban vs. placebo].

**Results:**

Among the 8,741 stented patients, 155 ST events (2.25%) occurred by Day 720. The cumulative incidences of early, late, and very late ST were 0.80%, 0.81%, and 0.77%, respectively. After multivariable adjustment, age ≥ 75 [hazard ratio (HR) = 2.13 (95% confidence interval, CI: 1.26–3.60)], a history of prior MI [HR = 1.81 (95% CI: 1.22–2.68)], low hemoglobin level [HR = 2.34 (95% CI: 1.59–3.44)], and high WBC count [HR = 1.58 (95% CI: 1.02–2.46)] were associated with a greater risk of overall ST, whereas DES [HR = 0.56 (95% CI: 0.38–0.83)] and rivaroxaban therapy [HR = 0.63 (95% CI: 0.44–0.88)] were associated with a lower risk of overall ST up to 720 days. Low hemoglobin level and high WBC count were associated with early ST (low hemoglobin: HR = 2.35 [95% CI: 1.34–4.12]; high WBC count: HR = 2.11 [95% CI: 1.17–3.81]). Low hemoglobin level and prior MI were associated with a greater risk of late ST (low hemoglobin: HR = 2.32 [95% CI: 1.26–4.27]; prior MI: HR = 2.98 [95% CI: 1.67–5.31]), whereas DES was associated with a lower risk of late ST [HR = 0.33 (95% CI: 0.16–0.67)]. Age ≥75 years was associated with very late ST.

**Conclusion:**

The study identified positive and negative associations with early, late, and very late ST. These variables may be useful in constructing risk assessment models for ST.

**Clinical Trial Registration:**

http://www.clinicaltrials.gov, identifier NCT00809965.

## Introduction

Stent thrombosis (ST) is a rare but potentially catastrophic complication of stent implantation that carries a high risk of mortality (1). It typically presents as myocardial infarction (MI), usually with ST segment elevation MI (STEMI), or death (2). Dual antiplatelet therapy (DAPT) has been demonstrated to prevent ST in patients with acute coronary syndrome (ACS) undergoing percutaneous coronary intervention (PCI) (3). The current guidelines from the American College of Cardiology (ACC)/American Heart Association (AHA) include a Class I recommendation to continue DAPT for at least 6 months after revascularization for stable ischemic heart disease and at least 12 months after an ACS (4). Prolonged DAPT beyond 6–12 months was placed as a Class IIb recommendation and may be considered based on the assessment of ischemic-bleeding tradeoff (4).

There is a pressing need to understand better the factors associated with increased risk of ST. Several clinical factors (e.g., ACS presentation, diabetes mellitus, current cigarette smoking, and reduced left ventricular ejection fraction) and procedural characteristics (e.g., stent undersizing, stent underdeployment, small stent diameter, greater stent length, and bifurcation stents) have been identified as predictors of ST (5–7). Yet, little is known about how laboratory tests may assist in stratifying ACS patients according to their ST risk. Few studies have assessed the prognostic significance of hemoglobin values and platelet counts in predicting ST (8, 9). However, it remains uncertain whether elevations in cardiac biomarkers or inflammatory markers during the index ACS event are linked to subsequent ST risk. As such, this study aimed to validate the association of hemoglobin and platelet count with the risk of ST in an international, multicenter Phase 3 trial with a relatively large sample size. Furthermore, the study sought to extend previous findings by investigating the predictive value of other available laboratory measurements, including cardiac biomarkers and white blood cell (WBC) counts (as a marker for inflammation), in relation to ST. In addition, due to the low event rate of ST, risk factors of ST based on the timing [i.e., early ST (within 30 days of implantation), late ST (between 1 month and 1 year from stent implantation), or very late ST (more than 1 year after stent implantation)] have not been well characterized in prior studies. As the underlying mechanisms of ST may differ across various periods (10), this substudy explored factors associated with early, late, and very late ST, including patient characteristics, laboratory tests, and treatments in the ATLAS ACS 2-TIMI 51 trial (A Randomized, Double-Blind, Placebo-Controlled, Event-Driven Multicenter Study to Evaluate the Efficacy and Safety of Rivaroxaban in Subjects With a Recent Acute Coronary Syndrome).

## Methods

The rationale and design of the ATLAS ACS 2-TIMI 51 trial (National Clinical Trial Identifier Number: NCT00809965) have been described previously (11, 12). In brief, the ATLAS ACS 2-TIMI 51 trial enrolled patients (≥18 years of age) who presented with symptoms suggestive of ACS. Enrollment occurred within 7 days of hospital admission, and patients were randomly assigned in a 1:1:1 fashion to twice-daily administration of either 2.5 or 5.0 mg of rivaroxaban or a placebo. Patients received the first dose of the study drug as soon as possible after randomization, but no sooner than 4 h after the final dose of intravenous unfractionated heparin, 2 h after the final dose of bivalirudin, and 12 h after the final dose of other intravenous or subcutaneous anticoagulants (e.g., enoxaparin or fondaparinux). All patients received standard medical therapy, including low-dose aspirin and a thienopyridine (either clopidogrel or ticlopidine) at a dose recommended according to the national or local guidelines. The patients were seen at 4 weeks, 12 weeks, and after that every 12 weeks, with a maximum follow-up of 31 months. The trial was conducted from November 26, 2008, to September 19, 2011. The primary efficacy endpoint of the ATLAS ACS 2 TIMI 51 trial was a composite of death from cardiovascular causes, MI, and stroke. ST was a predefined, independently adjudicated endpoint classified as definite, probable, or possible based on the Academic Research Consortium (ARC) definitions (13). The endpoint of the present analysis was ST (including definite, probable, and possible ST).

The present analysis was restricted to patients who received stent placement during the primary PCI for the index ACS. Patients with a history of coronary stent implantation were excluded from the analysis, because the specific date of their prior stent placement was not recorded, and it was not possible to accurately assess the time elapsed from stent placement to the occurrence of ST. The analysis was performed using a modified intention-to-treat (mITT) approach, described previously (12). A positive cardiac biomarker was defined as either a troponin level above the 99th percentile or a creatine kinase myocardial band above the upper limit of the normal range (according to the local laboratories), or both. The normal hemoglobin ranges were 12.5–17.0 g/dl for men and 11.5–15.8 g/dl for women. The normal ranges of platelet count and WBC count were 130–394 × 10^9^/L and 3.8–10.7 × 10^9^/L, respectively.

### Statistical analysis

The analyses in this substudy were *post hoc* and exploratory. Baseline characteristics of study participants stratified by treatment allocation (rivaroxaban 2.5 mg twice daily vs. rivaroxaban 5 mg twice daily vs. placebo) were compared using the chi-squared test for categorical variables and the one-way ANOVA or Kruskal–Wallis test for continuous variables, as appropriate.

The cumulative incidence of ST was assessed by Kaplan–Meier estimates at 30 days (early ST), 31–360 days (late ST), 361–720 days (very late ST), and up to 720 days, with Day 1 defined as the day of stent implantation. In addition, univariable and multivariable associations with ST were explored by fitting a Cox proportional hazards model. Candidate variables included patient characteristics (i.e., age, sex, ACS presentation, history of hypertension, smoking, diabetes mellitus, prior MI, congestive heart failure, prior ischemic stroke, and cancer), baseline laboratory tests (i.e., positive cardiac biomarker, hemoglobin, platelet count, WBC count), and treatment (i.e., the type of stent and anticoagulant with rivaroxaban vs. placebo). In addition, landmark analyses with the landmark time of 30, 31–360, and 361–720 days were performed to assess the association with early, late, and very late ST, respectively.

A nominal threshold of *P* < 0.05 was used to determine the significance of the association. All analyses were performed with SAS software version 9.4 (SAS Institute, Inc., NC, United States).

## Results

### Patient characteristics

Of the 15,342 patients included in the mITT analysis, 8,741 (57.0%) patients received coronary stent placement during PCI for the index ACS. Among those, 2,932 (33.5%) patients were assigned to receive rivaroxaban 2.5 mg twice daily, 2,910 (33.3%) were assigned to receive rivaroxaban 5 mg twice daily, and 2,899 (33.2%) were assigned to receive a placebo. As provided in Table 1, patient characteristics were generally balanced across patients with different treatment allocations, except that the mean age in the rivaroxaban arms was higher, the proportion of patients receiving DAPT differed, and the mean platelet level in the placebo arm was higher. Most patients received DAPT with low-dose aspirin plus a thienopyridine (99.7%) and statin (95.9%). Approximately 59.9%, 26.4%, and 13.7% of the patients presented with STEMI, non-ST segment elevation myocardial infarction (NSTEMI), and unstable angina, respectively. A total of 3,458 (39.6%) received drug-eluting stent (DES) implantation, and 5,283 (60.4%) received bare-metal stent (BMS) implantation. The rates of thrombolysis in myocardial infarction (TIMI) major bleeding not related to coronary artery bypass graft surgery (CABG) were 1.73%, 1.71%, and 0.52% in patients who received rivaroxaban 2.5 mg twice daily, rivaroxaban 5 mg twice daily, and placebo, respectively. The rates of TIMI minor bleeding were 0.80%, 1.22%, and 0.49%, respectively. The rates of bleeding event requiring medical attention were 11.14%, 14.10%, and 6.54%, respectively.

**Table 1:** Baseline characteristics of patients who received stent placement during index ACS.

Characteristic	Rivaroxaban2.5 mg twice daily (*N* = 2,932)	Rivaroxaban 5 mg twice daily (*N* = 2,910)	Placebo (*N* = 2,899)	*P*-value
Age (years), mean (SD)	61.8 (8.8)	61.9 (8.6)	61.3 (8.8)	0.007
Male sex, *n* (%)	2,321 (79.2)	2,291 (78.7)	2,277 (78.5)	0.84
Race, *n* (%)				0.57
White	2,215 (75.5)	2,207 (75.8)	2,219 (76.5)	
Asian	575 (19.6)	548 (18.8)	540 (18.6)	
Black/African American	18 (0.6)	19 (0.7)	20 (0.7)	
Others	124 (4.2)	136 (4.7)	118 (4.1)	
Body mass index (kg/m^2^), mean (SD)	27.8 (4.8)	27.7 (4.6)	27.7 (4.5)	0.28
Creatinine clearance (ml/min), *n* (%)				0.67
>80	1,791 (62.0)	1,767 (61.7)	1,798 (62.9)	
≥50–≤80	945 (32.7)	964 (33.7)	922 (32.3)	
≥30–<50	148 (5.1)	124 (4.3)	131 (4.6)	
<30	5 (0.2)	8 (0.3)	6 (0.2)	
Antiplatelet therapy, *n* (%)				0.026
Aspirin plus clopidogrel or ticlopidine	2,920 (99.6)	2,908 (99.9)	2,888 (99.6)	
Aspirin	12 (0.4)	2 (0.1)	11 (0.4)	
ACS presentation, *n* (%)				0.87
STEMI	1,757 (59.9)	1,730 (59.5)	1,749 (60.3)	
NSTEMI	761 (26.0)	779 (26.8)	764 (26.4)	
Unstable angina	414 (14.1)	401 (13.8)	386 (13.3)	
Stent type, *n* (%)				0.24
DES	1,128 (38.5)	1,152 (39.6)	1,178 (40.6)	
BMS	1,804 (61.5)	1,758 (60.4)	1,721 (59.4)	
Medical history, *n* (%)				
Hypertension	1,824 (62.2)	1,809 (62.2)	1,782 (61.5)	0.82
Smoking	1,298 (44.3)	1,227 (42.2)	1,269 (43.8)	0.24
Diabetes mellitus	903 (30.8)	877 (30.1)	870 (30.0)	0.78
Myocardial infarction	575 (19.6)	602 (20.7)	597 (20.6)	0.53
Congestive heart failure	126 (4.3)	138 (4.7)	129 (4.4)	0.71
Ischemic stroke	28 (1.0)	31 (1.1)	23 (0.8)	0.56
Cancer	100 (3.4)	105 (3.6)	101 (3.5)	0.92
Chronic kidney disease	64 (2.2)	73 (2.5)	70 (2.4)	0.70
Atrial fibrillation	19 (0.6)	23 (0.8)	28 (1.0)	0.39
Medications, *n* (%)				
ACEI or ARB	1,381 (47.1)	1,367 (47.0)	1,379 (47.6)	0.89
Beta blocker	2,134 (72.8)	2,107 (72.4)	2,130 (73.5)	0.65
Calcium channel blocker	669 (22.8)	617 (21.2)	606 (20.9)	0.16
Statin	2,807 (95.7)	2,791 (95.9)	2,788 (96.2)	0.70
Positive cardiac biomarker, *n* (%)	2,639 (90.0)	2,609 (89.7)	2,629 (90.7)	0.43
Hemoglobin (g/dl), mean (SD)	12.75 (3.58)	12.68 (3.71)	12.69 (3.80)	0.75
Hemoglobin category, *n* (%)				0.50
High	19 (0.7)	15 (0.6)	17 (0.6)	
Normal	2,250 (83.7)	2,271 (85.1)	2,278 (85.4)	
Low	418 (15.6)	382 (14.3)	374 (14.0)	
Platelet (×10^9^/L), mean (SD)	247.9 (68.9)	246.1 (68.4)	251.1 (73.2)	0.034
Platelet category, *n* (%)				0.48
High	77 (2.9)	66 (2.5)	87 (3.3)	
Normal	2,521 (95.0)	2,523 (95.5)	2,494 (94.8)	
Low	57 (2.1)	52 (2.0)	50 (1.9)	
White blood cell (×10^9^/L), mean (SD)	8.1 (2.3)	8.1 (2.4)	8.2 (2.3)	0.76
White blood cell category, *n* (%)				0.43
High	324 (12.1)	325 (12.2)	336 (12.6)	
Normal	2,350 (87.5)	2,325 (87.1)	2,309 (86.5)	
Low	13 (0.5)	18 (0.7)	24 (0.9)	

ARB, angiotensin II receptor blocker; SD, standard deviation.

**Figure 1:**
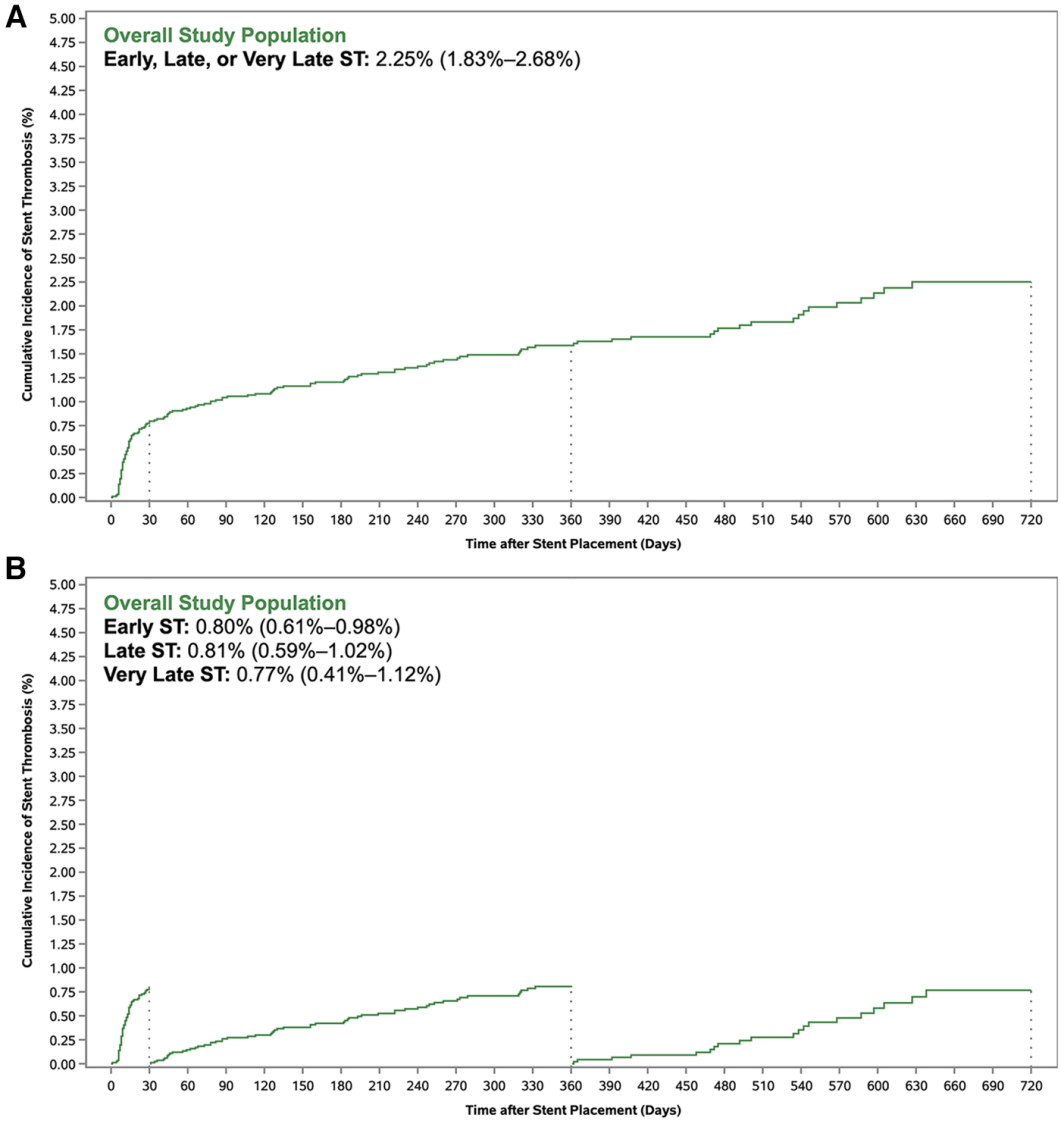
Cumulative incidence of stent thrombosis with (**A**) standard Kaplan–Meier method and (**B**) landmark analysis.

A total of 155 ST events occurred by Day 720, including 72 early ST events (within 30 days), 62 late ST events (from 31 to 360 days), and 21 very late ST events (from 361 to 720 days). Of the 155 ST events, there were 75 categorized as definite ST, 41 as probable ST, and 38 as possible ST (with one ST event remaining uncategorized). A total of 129 patients had only one ST event (64, 48, and 17 were early, late, and very late ST events, respectively), and 13 patients had two ST events (8, 14, and 4 were early, late, and very late ST events, respectively). The cumulative incidence of overall ST at 720 days was 2.25% [95% confidence interval (CI): 1.83%–2.68%] (Figure 1A). The cumulative incidence of early, late, and very late ST was 0.80% (95% CI: 0.61%–0.98%), 0.81% (95% CI: 0.59%–1.02%), and 0.77% (95% CI: 0.41%–1.12%), respectively (Figure 1B).

### Factors associated with stent thrombosis up to 720 days

In univariable analysis, the factors associated with ST up to 720 days included age ≥ 75 years, a history of prior MI, congestive heart failure, low hemoglobin level, high platelet count, high WBC count, DES, and rivaroxaban therapy (Table 2). After multivariable adjustment, age ≥ 75 years [hazard ratio (HR) = 2.127 (95% CI: 1.257–3.598)], a history of prior MI [HR = 1.806 (95% CI: 1.216–2.682)], low hemoglobin level [HR = 2.339 (95% CI: 1.590–3.440)], and high WBC count [HR = 1.583 (95% CI: 1.017–2.464)] remained associated with a greater risk of ST up to 720 days, whereas DES [HR = 0.563 (95% CI: 0.380–0.833)] and rivaroxaban therapy [HR = 0.625 (95% CI: 0.443–0.882)] were associated with a lower risk of ST up to 720 days. Hemoglobin or WBC category did not significantly modify the effect of rivaroxaban on the risk of ST (interaction *P* = 0.95 and *P* = 0.98, respectively).

**Table 2:** Factors associated with stent thrombosis (up to 720 days).

Variable	ST (up to 720 days)			
	Univariable analysis		Multivariable analysis	
	HR (95% CI)	*P-*value	HR (95% CI)	*P*-value
Patient characteristic				
Age (≥75 vs. <75 years)	1.932 (1.192–3.133)	0.0075	2.127 (1.257–3.598)	0.0049
Sex (males vs. females)	1.001 (0.669–1.498)	0.9958	0.987 (0.644–1.512)	0.9515
ACS presentation (MI vs. unstable angina)	1.739 (0.963–3.142)	0.0666	1.156 (0.477–2.800)	0.7488
Hypertension	0.835 (0.598–1.165)	0.2878	0.735 (0.510–1.059)	0.0983
Smoking	1.346 (0.969–1.871)	0.0766	1.362 (0.952–1.949)	0.0906
Diabetes mellitus	1.197 (0.847–1.692)	0.3077	1.415 (0.980–2.043)	0.0644
Prior myocardial infarction	1.641 (1.144–2.353)	0.0071	1.806 (1.216–2.682)	0.0034
Congestive heart failure	2.201 (1.244–3.892)	0.0067	1.739 (0.942–3.212)	0.0771
Prior ischemic stroke	1.716 (0.425–6.930)	0.4485	1.704 (0.416–6.975)	0.4583
Cancer	1.010 (0.414–2.465)	0.9827	1.082 (0.440–2.660)	0.8639
Laboratory test				
Cardiac biomarker (positive vs. negative)	2.132 (0.997–4.558)	0.0508	2.095 (0.657–6.680)	0.2111
Hemoglobin (low vs. normal)	2.412 (1.663–3.498)	<0.0001	2.339 (1.590–3.440)	<0.0001
Platelet (high vs. normal)	2.113 (1.034–4.319)	0.0403	1.654 (0.801–3.417)	0.1742
White blood cell (high vs. normal)	1.865 (1.230–2.828)	0.0033	1.583 (1.017–2.464)	0.0419
Treatment				
Stent type (DES vs. BMS)	0.559 (0.384–0.813)	0.0024	0.563 (0.380–0.833)	0.0041
Anticoagulant therapy (rivaroxaban vs. placebo)	0.653 (0.468–0.909)	0.0117	0.625 (0.443–0.882)	0.0075

### Factors associated with early stent thrombosis

In univariable analysis, the factors associated with early ST (within 30 days) included low hemoglobin level, high WBC count, and DES (Table 3). After multivariable adjustment, only low hemoglobin level [HR = 2.353 (95% CI: 1.344–4.120)] and high WBC count [HR = 2.108 (95% CI: 1.167–3.809)] remained associated with early ST.

**Table 3:** Factors associated with early stent thrombosis (≤30 days).

Variable	Early ST (≤30 days)			
	Univariable analysis		Multivariable analysis	
	HR (95% CI)	*P*-value	HR (95% CI)	*P*-value
Patient characteristic				
Age (≥75 vs. <75 years)	1.527 (0.731–3.191)	0.2603	2.074 (0.944–4.555)	0.0692
Sex (males vs. females)	1.156 (0.632–2.113)	0.6384	1.169 (0.610–2.242)	0.6374
ACS presentation (MI vs. unstable angina)	2.045 (0.823–5.082)	0.1233	1.272 (0.325–4.980)	0.7298
Hypertension	0.708 (0.441–1.137)	0.1534	0.690 (0.408–1.169)	0.1682
Smoking	1.504 (0.937–2.414)	0.0908	1.467 (0.867–2.482)	0.1533
Diabetes mellitus	1.386 (0.852–2.256)	0.1885	1.687 (0.997–2.855)	0.0513
Prior myocardial infarction	1.181 (0.675–2.066)	0.5590	1.340 (0.721–2.490)	0.3540
Congestive heart failure	1.315 (0.479–3.608)	0.5949	1.331 (0.472–3.759)	0.5890
Prior ischemic stroke	1.581 (0.220–11.389)	0.6491	1.684 (0.230–12.315)	0.6079
Cancer	1.253 (0.394–3.985)	0.7021	1.465 (0.453–4.733)	0.5234
Laboratory test				
Cardiac biomarker (positive vs. negative)	2.408 (0.757–7.656)	0.1366	1.566 (0.282–8.689)	0.6082
Hemoglobin (low vs. normal)	2.402 (1.408–4.100)	0.0013	2.353 (1.344–4.120)	0.0027
Platelet (high vs. normal)	2.262 (0.821–6.229)	0.1144	1.712 (0.614–4.776)	0.3045
White blood cell (high vs. normal)	2.532 (1.456–4.402)	0.0010	2.108 (1.167–3.809)	0.0135
Treatment				
Stent type (DES vs. BMS)	0.582 (0.343–0.988)	0.0448	0.632 (0.363–1.100)	0.1048
Anticoagulant therapy (rivaroxaban vs. placebo)	0.684 (0.424–1.103)	0.1191	0.629 (0.380–1.040)	0.0706

### Factors associated with late stent thrombosis

In univariable analysis, the factors associated with late ST (31–360 days) included age ≥75 years, a history of prior MI, congestive heart failure, low hemoglobin level, and DES (Table 4). After multivariable adjustment, prior MI [HR = 2.978 (95% CI: 1.671–5.309)] and low hemoglobin level [HR = 2.315 (95% CI: 1.256–4.265)] remained associated with late ST, whereas DES [HR = 0.334 (95% CI: 0.165–0.673)] was protective against late ST.

**Table 4:** Factors associated with late stent thrombosis (31–360 days).

Variable	Late ST (31–360 days)			
	Univariable analysis		Multivariable analysis	
	HR (95% CI)	*P*-value	HR (95% CI)	*P*-value
Patient characteristic				
Age (≥75 vs. <75 years)	2.180 (1.031–4.609)	0.0412	1.919 (0.831–4.432)	0.1271
Sex (males vs. females)	0.972 (0.513–1.840)	0.9297	0.916 (0.471–1.783)	0.7964
ACS presentation (MI vs. unstable angina)	1.307 (0.560–3.048)	0.5356	0.814 (0.249–2.659)	0.7332
Hypertension	0.881 (0.517–1.500)	0.6400	0.696 (0.391–1.237)	0.2168
Smoking	1.132 (0.670–1.915)	0.6426	1.157 (0.661–2.026)	0.6087
Diabetes mellitus	0.987 (0.558–1.744)	0.9628	1.192 (0.656–2.163)	0.5642
Prior myocardial infarction	2.774 (1.629–4.724)	0.0002	2.978 (1.671–5.309)	0.0002
Congestive heart failure	3.202 (1.450–7.069)	0.0040	1.941 (0.804–4.682)	0.1402
Prior ischemic stroke	2.584 (0.358–18.680)	0.3468	2.301 (0.307–17.235)	0.4173
Cancer	0.512 (0.071–3.700)	0.5071	0.554 (0.076–4.027)	0.5593
Laboratory test				
Cardiac biomarker (positive vs. negative)	1.919 (0.600–6.141)	0.2721	4.084 (0.689–24.202)	0.1212
Hemoglobin (low vs. normal)	2.304 (1.273–4.171)	0.0058	2.315 (1.256–4.265)	0.0071
Platelet (high vs. normal)	2.734 (0.987–7.576)	0.0531	2.191 (0.772–6.216)	0.1405
White blood cell (high vs. normal)	1.444 (0.706–2.953)	0.3146	1.175 (0.547–2.523)	0.6793
Treatment				
Stent type (DES vs. BMS)	0.349 (0.176–0.691)	0.0025	0.334 (0.165–0.673)	0.0022
Anticoagulant therapy (rivaroxaban vs. placebo)	0.634 (0.374–1.073)	0.0897	0.616 (0.359–1.058)	0.0791

### Factors associated with very late stent thrombosis

In univariable analysis, the factors associated with very late ST (361–720 days) included age ≥75 years and low hemoglobin level (Table 5). After multivariable adjustment, only age ≥75 years [HR = 3.710 (95% CI: 1.116–12.330)] remained associated with very late ST.

**Table 5:** Factors associated with very late stent thrombosis (361–720 days).

Variable	Very late ST (361–720 days)			
	Univariable analysis		Multivariable analysis	
	HR (95% CI)	*P-*value	HR (95% CI)	*P-*value
Patient characteristic				
Age (≥75 vs. <75 years)	3.653 (1.221–10.929)	0.0205	3.710 (1.116–12.330)	0.0324
Sex (males vs. females)	0.516 (0.206–1.293)	0.1581	0.507 (0.190–1.351)	0.1746
ACS presentation (MI vs. unstable angina)	3.268 (0.437–24.415)	0.2484	13.822 (0.057–3,358.386)	0.3487
Hypertension	1.114 (0.445–2.793)	0.8177	0.892 (0.335–2.371)	0.8188
Smoking	1.624 (0.673–3.920)	0.2804	2.027 (0.776–5.289)	0.1490
Diabetes mellitus	1.486 (0.607–3.634)	0.3859	1.781 (0.694–4.571)	0.2299
Prior myocardial infarction	0.910 (0.304–2.723)	0.8663	1.025 (0.320–3.279)	0.9670
Congestive heart failure	2.397 (0.556–10.336)	0.2409	1.940 (0.412–9.143)	0.4021
Prior ischemic stroke	—	0.9897	—	0.9966
Cancer	1.405 (0.188–10.501)	0.7401	1.203 (0.157–9.209)	0.8587
Laboratory test				
Cardiac biomarker (positive vs. negative)	2.253 (0.302–16.831)	0.4285	0.178 (0.001–42.905)	0.5377
Hemoglobin (low vs. normal)	2.827 (1.074–7.437)	0.0353	2.278 (0.837–6.200)	0.1070
Platelet (high vs. normal)	—	0.9920	—	0.9932
White blood cell (high vs. normal)	0.786 (0.182–3.402)	0.7424	0.779 (0.177–3.425)	0.7408
Treatment				
Stent type (DES vs. BMS)	1.193 (0.488–2.919)	0.6991	1.061 (0.408–2.760)	0.9026
Anticoagulant therapy (rivaroxaban vs. placebo)	0.800 (0.327–1.956)	0.6244	0.863 (0.338–2.201)	0.7574

### Summary of factors associated with stent thrombosis

**Figure 2:**
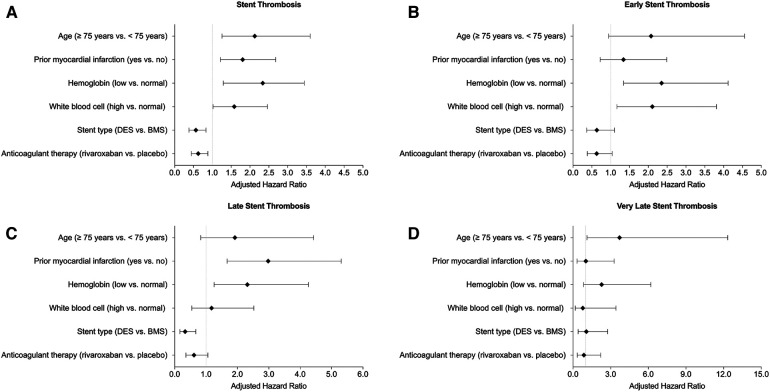
Summary of association with (**A**) all stent thrombosis, (**B**) early stent thrombosis, (**C**) late stent thrombosis, and (**D**) very late stent thrombosis.

The results of independent factors with positive (including age ≥75 years, a history of prior MI, low hemoglobin level, and high WBC count) and negative (including DES and rivaroxaban) association with ST up to 720 days (Panel A), early ST (Panel B), late ST (Panel C), and very late ST (Panel D) were summarized in Figure 2.

With respect to factors with a positive association, high WBC count was predictive of ST up to 720 days and early ST. Low hemoglobin level was predictive of ST up to 720 days, early ST, and late ST. Prior MI was predictive of ST up to 720 days and late ST. Age ≥75 years was predictive of ST up to 720 days and very late ST. With respect to factors with a negative association, DES was protective against ST up to 720 days and late ST. Rivaroxaban therapy was protective against ST for up to 720 days.

Landmark analyses of the six factors were provided in Supplementary Figures S1–S6. Compared with patients aged <75 years, those aged ≥75 years had a significantly greater risk of late and very late ST (Supplementary Figure S1). The association between prior MI and ST was primarily driven by the substantial risk difference from 31 to 360 days (i.e., late ST) (Supplementary Figure S2). Compared with patients with normal hemoglobin levels, anemic patients had a significantly greater ST risk within the first year (i.e., early and late ST) (Supplementary Figure S3). Patients with leukocytosis had a significantly greater risk for ST only within the first 30 days following stent implantation (i.e., early ST) (Supplementary Figure S4). Compared with BMS, DES was associated with a significantly reduced ST risk within the first year, encompassing early and late ST events. During the second year, there was no significant difference in ST risk between DES and BMS (Supplementary Figure S5). While patients who received rivaroxaban had a reduced ST risk at 2 years, its effect on early, late, or very late ST was not statistically significant (Supplementary Figure S6).

## Discussion

In the ATLAS ACS 2-TIMI 51 trial, the incidence of early, late, and very late ST was 0.80%, 0.81%, and 0.77% among patients with a recent ACS. These findings were consistent with prior studies, which reported an incidence of <1% for early ST, 0.5%–1.0% for late ST, and 0.2%–0.4% for very late ST (5). While confirming the established factors associated with ST (such as advanced age, prior MI, and DES), the study extends the current knowledge by demonstrating the usefulness of commonly available blood tests, including WBC and hemoglobin, in predicting early and late ST. In a patient-level pooled analysis of ACS patients undergoing PCI with stent implantation from the HORIZON-AMI and ACUITY trials, Dangas et al. developed a 1-year ST risk score that included type of ACS, smoking status, insulin-treated diabetes mellitus, history of PCI, baseline platelet count, absence of early heparin use, aneurysm or ulceration, baseline and final TIMI flow grade, and the number of vessels treated (9). While we did not find an association between ST and the type of ACS, smoking status, or platelet count, we observed that high WBC counts and low hemoglobin levels were associated with a greater ST risk. As part of the standard measures of complete blood count by automated instruments in the hematology laboratory, WBC and hemoglobin are of low cost and can be readily incorporated into ST risk assessment.

The data suggested that a high WBC count was independently associated with an increased risk of ST by 30 days. Previous analysis of the ATLAS ACS 2-TIMI 51 trial indicates an association between ischemic events and high WBC count (14–16). The plausible mechanisms for such association included alteration of platelet function, tissue factor formation, endothelial injury, and extrinsic pathway activation (14). Specifically, these mechanisms can be classified into two major categories: (1) mechanisms that directly involve WBC in the development of ischemic events: (i) direct effect of WBC surface antigen (CD11b/CD18) on activation of the coagulation cascade and the subsequent plaque formation (17), (ii) plugging capillary lumen caused by high viscosity, and (iii) platelet-WBC aggregation caused by WBC surface antigens (18); (2) mechanisms that indirectly involve WBCs as an index of inflammation and coagulation: (i) decreased epicardial and myocardial perfusion (19) and (ii) endothelial injury due to release of oxygen-free radicals and other factors that may promote complement activation, cause reperfusion injury, and provoke an inflammatory response, which may persist after the resolution of the acute event and last up to 6 months (20, 21).

In agreement with previous studies, the present study indicated that low hemoglobin levels were associated with an increased risk of ST (8, 22, 23). While the exact mechanisms remain unclear, anemic patients may be more likely to experience thrombotic events (24, 25). The presence of anemia might reflect an underlying condition that predisposes patients to thrombosis, such as iron deficiency, chronic inflammation, malignancy, and chronic kidney disease (26–28). For instance, studies have suggested that lower hemoglobin and serum iron levels are associated with elevated levels of coagulation factor VIII and accelerated platelet aggregation, which can contribute to ST (29, 30). Therefore, low hemoglobin levels could be considered an integrative marker of multiple pathologic processes in ACS patients. Another possible explanation is that anemia may be associated with endothelial dysfunction, blood stasis, or a hypercoagulable state, leading to a greater risk of thrombus formation (31–33).

Previous data have confirmed the superiority of second-generation DES over BMS in terms of the risk of ST at 1 year (34, 35). A pooled analysis of data from recent trials with 26,616 patients comparing the outcomes after implantation of new-generation DES vs. BMS over an average follow-up of 3.2 years demonstrated lower definite ST with DES [HR = 0.63 (95% CI: 0.50–0.80), *P* < 0.001] (35). This is similar to our findings that showed a lower risk of ST with DES over a follow-up period of 2 years [HR = 0.563 (95% CI) 0.380–0.833], *P* = 0.004). However, Brodie et al. (36) demonstrated DES as the only protective factor of ST (HR = 3.73, 95% CI: 1.81–7.88). It is worth mentioning that Brodie et al. compared the first-generation DES with BMS. The first-generation DES had been associated with higher rates of late and very late ST, with a reported very late ST rate of up to 2% with first-generation stents as compared with 0.2%–0.4% per year with newer DES (5). The higher thrombosis risk resulted in the discontinuation of first-generation DES and the development of the new generation of DES (37). The mechanisms of newer generation DES and the reduction of ST are not well understood. Some investigators argued that this reduction occurs due to a more extended period of DAPT with the application of DES. However, the LEADERS FREE trial demonstrated that ST might occur irrespective of DAPT duration. In the LEADERS FREE trial, patients at high risk of bleeding were randomized to receive either DES or BMS, and 1-month DAPT was applied irrespective of stent type. The trial found a significantly lower rate of ST with DES compared to BMS at 30 days and over a period of 390 days (38). The protection against late ST by DES may be due to the effect of delayed endothelialization of the coronary lumen by DES-released antiproliferative drugs (10). There is no clear consensus on whether DES is more effective than BMS beyond 1 year (35). In the present analysis, DES usage was associated with a lower risk of overall ST up to 720 days, primarily attributed to a reduction in late ST events. Nonetheless, we did not observe a significant difference in the risk of very late ST between DES and BMS usage [HR = 1.061 95% CI: (0.408–2.760), *P* = 0.9026]. The lack of a discernible advantage with DES could potentially be influenced by the prolonged duration of DAPT, typically spanning a minimum of 6–12 months, in patients who underwent DES implantation. Future studies are warranted to focus on reducing thrombotic events beyond 1 year.

In this analysis, patients 75 years or older were at a higher risk of developing very late ST. There have been mixed results with respect to whether younger or older age was associated with an increased risk of ST. In a study conducted among 124 patients with ST, a 1-year decrease in age was associated with an 8% increase in very late ST (39). Two previous reports also suggested younger age as a predictor of ST (40, 41). However, these studies were performed when first-generation DES was used, which may have affected the results. It is, therefore, not known whether the association of younger age with increased risk of ST applies to the newer generation of DES. Of note, it has been demonstrated that premature discontinuation of P2Y12 inhibitors was associated with older age, which may help explain the higher ST risk (42). In addition, consistent with previous reports, a history of previous MI was another predictor of late ST in this analysis (36). Older patients are more likely to have a complicated cardiac history, including previous MI, thus contributing to an increased ST risk.

## Limitations

The current findings should be interpreted in the context of several limitations. First, only 155 adjudicated ST events occurred through the end of follow-up by 720 days, and the landmark analysis may be underpowered. Second, the maximum follow-up was 31 months, which may be inadequate for analyzing very late ST. Third, although the majority (99.7%) of patients received DAPT therapy at baseline, information about DAPT interruption was not available for analysis, which might have affected the precision of the estimates. It is important to emphasize that, although clopidogrel remains commonly used as part of the DAPT regimen, contemporary clinical practice guidelines now recommend more potent P2Y12 inhibitors, such as prasugrel and ticagrelor. This recommendation stems from head-to-head comparisons in recent clinical trials, demonstrating the superiority of these next-generation inhibitors over clopidogrel in preventing ischemic events. Furthermore, recent evidence from the M.O.Ca. registry suggests a potential protective role of cangrelor in reducing in-hospital definite ST among ACS patients when administered in addition to the current standard-of-care P2Y12 inhibitors (43). Aside from antiplatelet therapy, the range in collection times for the various laboratory investigations was not controlled for in the study. Fourth, it is important to note that several established lesion-, device-, and procedure-related risk factors, such as angiographic characteristics, the type of DES employed, stent dimensions (diameter and length), the utilization of intracoronary imaging, and the practice of bifurcation stenting, were not included in the study due to unavailability. The potential complex interactions among these factors could significantly influence the timeframes of ST. It is essential to consider these limitations when interpreting the results of the current study. Fifth, in the ATLAS ACS 2-TIMI 51 trial, participants were enrolled within 7 days (with a mean ± SD of 1.5 ± 0.9 days) after being admitted to the hospital because of an acute coronary syndrome, once their condition had been stabilized and initial management strategies (e.g., revascularization) were completed. In this study, Day 1 was defined as the day of stent implantation, marking the initiation of stent exposure. This approach was chosen to accurately capture the timing when patients became at risk for ST, as opposed to using the day of randomization or treatment initiation in the main analysis. Nevertheless, the incidence of early ST might have been underestimated in this study, as acute ST events (i.e., ST events that occurred within 24 h of stent placement) prior to study enrollment were not accounted for. Sixth, to ensure a sufficient event frequency at each landmark time for exploring potential predictors in the multivariable analysis, we included definite, probable, and possible ST as the endpoint. Under the ARC classification scheme, definite ST exhibits a high level of specificity, but is prone to potentially underestimating the actual occurrence. Conversely, possible ST offers enhanced sensitivity, but lacks diagnostic certainty (44). It is important to note that incorporating possible ST into the analysis could have introduced some uncertainties, and the results from the exploratory analysis should be validated in future studies. Seventh, according to the practice guidelines, DES is preferred over BMS in patients undergoing PCI (45). While the study findings were consistent with the guideline recommendations, it should be highlighted that the role of BMS is limited in current practice. Eighth, it is important to consider the risk of overfitting in the multivariable analysis when examining the type of ST by timeframe. Last, this is a *post hoc* analysis of a subset of stented ACS patients who agreed to participate in a clinical trial with specific enrollment criteria. The findings from the present study do not suggest causality and should be considered exploratory and hypothesis-generating.

## Conclusion

The study identified factors associated with early ST (high WBC count and low hemoglobin), late ST (low hemoglobin, prior MI, and DES), very late ST (advanced age), and overall ST (advanced age, prior MI, low hemoglobin, high WBC, DES, and rivaroxaban). These variables may be useful in constructing risk assessment models for ST.

## Data Availability

The datasets presented in this article are not readily available because they contain restricted data. Requests to access the datasets should be directed to the corresponding author.
