# Supplementary material for: Factors associated with early, late, and very late stent thrombosis among patients with acute coronary syndrome undergoing coronary stent placement: analysis from the ATLAS ACS 2-TIMI 51 trial
Source: Front Cardiovasc Med. 2024 Jan 8;10:1269011. doi: 10.3389/fcvm.2023.1269011 (PMC10800486; doi:10.3389/fcvm.2023.1269011)

Supplementary Material

Factors Associated with Early, Late, and Very Late Stent Thrombosis Among Patients with Acute Coronary Syndrome Undergoing Coronary Stent Placement: Analysis from the ATLAS ACS 2–TIMI 51 Trial

Table S1. Factors associated with early stent thrombosis (≤ 30 days)

Table S2. Factors associated with late stent thrombosis (31 to 360 days)

Table S3. Factors associated with very late stent thrombosis (361 to 720 days)

Figure S1. Cumulative incidence of stent thrombosis stratified by age category (≥ 75 years vs. < 75 years): (A) standard Kaplan-Meier method, (B) landmark analysis

Figure S2. Cumulative incidence of stent thrombosis stratified by prior myocardial infarction: (A) standard Kaplan-Meier method, (B) landmark analysis

Figure S3. Cumulative incidence of stent thrombosis stratified by hemoglobin category (low vs. normal): (A) standard Kaplan-Meier method, (B) landmark analysis

Figure S4. Cumulative incidence of stent thrombosis stratified by white blood cell category (high vs. normal): (A) standard Kaplan-Meier method, (B) landmark analysis

Figure S5. Cumulative incidence of stent thrombosis stratified by stent type (drug-eluting stent vs. bare-metal stent): (A) standard Kaplan-Meier method, (B) landmark analysis

Figure S6. Cumulative incidence of stent thrombosis stratified by anticoagulant therapy (rivaroxaban vs. placebo): (A) standard Kaplan-Meier method, (B) landmark analysis

Supplementary Material

Table S1. Factors associated with early stent thrombosis (≤ 30 days)

Variable	Early ST (≤ 30 days)			
	Univariable Analysis		Multivariable Analysis	
	HR (95% CI)	P value	HR (95% CI)	P value
**Patient characteristic**				
Age (≥ 75 vs. < 75 years)	1.527 (0.731–3.191)	0.2603	2.074 (0.944–4.555)	0.0692
Sex (males vs. females)	1.156 (0.632–2.113)	0.6384	1.169 (0.610–2.242)	0.6374
ACS presentation (MI vs. unstable angina)	2.045 (0.823–5.082)	0.1233	1.272 (0.325–4.980)	0.7298
Hypertension	0.708 (0.441–1.137)	0.1534	0.690 (0.408–1.169)	0.1682
Smoking	1.504 (0.937–2.414)	0.0908	1.467 (0.867–2.482)	0.1533
Diabetes mellitus	1.386 (0.852–2.256)	0.1885	1.687 (0.997–2.855)	0.0513
Prior myocardial infarction	1.181 (0.675–2.066)	0.5590	1.340 (0.721–2.490)	0.3540
Congestive heart failure	1.315 (0.479–3.608)	0.5949	1.331 (0.472–3.759)	0.5890
Prior ischemic stroke	1.581 (0.220–11.389)	0.6491	1.684 (0.230–12.315)	0.6079
Cancer	1.253 (0.394–3.985)	0.7021	1.465 (0.453–4.733)	0.5234
**Laboratory test**				
Cardiac biomarker (positive vs. negative)	2.408 (0.757–7.656)	0.1366	1.566 (0.282–8.689)	0.6082
Hemoglobin (low vs. normal)	2.402 (1.408–4.100)	0.0013	2.353 (1.344–4.120)	0.0027
Platelet (high vs. normal)	2.262 (0.821–6.229)	0.1144	1.712 (0.614–4.776)	0.3045
White blood cell (high vs. normal)	2.532 (1.456–4.402)	0.0010	2.108 (1.167–3.809)	0.0135
**Treatment**				
Stent type (DES vs. BMS)	0.582 (0.343–0.988)	0.0448	0.632 (0.363–1.100)	0.1048
Anticoagulant therapy (rivaroxaban vs. placebo)	0.684 (0.424–1.103)	0.1191	0.629 (0.380–1.040)	0.0706

Abbreviations: ACS, acute coronary syndrome; BMS, bare-metal stent; CI, confidence interval; DES, drug-eluting stent; HR, hazard ratio; MI, myocardial infarction; NSTEMI, non-ST segment elevation myocardial infarction; ST, stent thrombosis; STEMI, ST segment elevation myocardial infarction.

Supplementary Material

Table S2. Factors associated with late stent thrombosis (31 to 360 days)

Variable	Late ST (31 to 360 Days)			
	Univariable Analysis		Multivariable Analysis	
	HR (95% CI)	P value	HR (95% CI)	P value
**Patient characteristic**				
Age (≥ 75 vs. < 75 years)	2.180 (1.031–4.609)	0.0412	1.919 (0.831–4.432)	0.1271
Sex (males vs. females)	0.972 (0.513–1.840)	0.9297	0.916 (0.471–1.783)	0.7964
ACS presentation (MI vs. unstable angina)	1.307 (0.560–3.048)	0.5356	0.814 (0.249–2.659)	0.7332
Hypertension	0.881 (0.517–1.500)	0.6400	0.696 (0.391–1.237)	0.2168
Smoking	1.132 (0.670–1.915)	0.6426	1.157 (0.661–2.026)	0.6087
Diabetes mellitus	0.987 (0.558–1.744)	0.9628	1.192 (0.656–2.163)	0.5642
Prior myocardial infarction	2.774 (1.629–4.724)	0.0002	2.978 (1.671–5.309)	0.0002
Congestive heart failure	3.202 (1.450–7.069)	0.0040	1.941 (0.804–4.682)	0.1402
Prior ischemic stroke	2.584 (0.358–18.680)	0.3468	2.301 (0.307–17.235)	0.4173
Cancer	0.512 (0.071–3.700)	0.5071	0.554 (0.076–4.027)	0.5593
**Laboratory test**				
Cardiac biomarker (positive vs. negative)	1.919 (0.600–6.141)	0.2721	4.084 (0.689–24.202)	0.1212
Hemoglobin (low vs. normal)	2.304 (1.273–4.171)	0.0058	2.315 (1.256–4.265)	0.0071
Platelet (high vs. normal)	2.734 (0.987–7.576)	0.0531	2.191 (0.772–6.216)	0.1405
White blood cell (high vs. normal)	1.444 (0.706–2.953)	0.3146	1.175 (0.547–2.523)	0.6793
**Treatment**				
Stent type (DES vs. BMS)	0.349 (0.176–0.691)	0.0025	0.334 (0.165–0.673)	0.0022
Anticoagulant therapy (rivaroxaban vs. placebo)	0.634 (0.374–1.073)	0.0897	0.616 (0.359–1.058)	0.0791

Abbreviations: ACS, acute coronary syndrome; BMS, bare-metal stent; CI, confidence interval; DES, drug-eluting stent; HR, hazard ratio; MI, myocardial infarction; NSTEMI, non-ST segment elevation myocardial infarction; ST, stent thrombosis; STEMI, ST segment elevation myocardial infarction.

Supplementary Material

Table S3. Factors associated with very late stent thrombosis (361 to 720 days)

Variable	Very Late ST (361 to 720 Days)			
	Univariable Analysis		Multivariable Analysis	
	HR (95% CI)	P value	HR (95% CI)	P value
**Patient characteristic**				
Age (≥ 75 vs. < 75 years)	3.653 (1.221–10.929)	0.0205	3.710 (1.116–12.330)	0.0324
Sex (males vs. females)	0.516 (0.206–1.293)	0.1581	0.507 (0.190–1.351)	0.1746
ACS presentation (MI vs. unstable angina)	3.268 (0.437–24.415)	0.2484	13.822 (0.057–3358.386)	0.3487
Hypertension	1.114 (0.445–2.793)	0.8177	0.892 (0.335–2.371)	0.8188
Smoking	1.624 (0.673–3.920)	0.2804	2.027 (0.776–5.289)	0.1490
Diabetes mellitus	1.486 (0.607–3.634)	0.3859	1.781 (0.694–4.571)	0.2299
Prior myocardial infarction	0.910 (0.304–2.723)	0.8663	1.025 (0.320–3.279)	0.9670
Congestive heart failure	2.397 (0.556–10.336)	0.2409	1.940 (0.412–9.143)	0.4021
Prior ischemic stroke	–	0.9897	–	0.9966
Cancer	1.405 (0.188–10.501)	0.7401	1.203 (0.157–9.209)	0.8587
**Laboratory test**				
Cardiac biomarker (positive vs. negative)	2.253 (0.302–16.831)	0.4285	0.178 (0.001–42.905)	0.5377
Hemoglobin (low vs. normal)	2.827 (1.074–7.437)	0.0353	2.278 (0.837–6.200)	0.1070
Platelet (high vs. normal)	–	0.9920	–	0.9932
White blood cell (high vs. normal)	0.786 (0.182–3.402)	0.7424	0.779 (0.177–3.425)	0.7408
**Treatment**				
Stent type (DES vs. BMS)	1.193 (0.488–2.919)	0.6991	1.061 (0.408–2.760)	0.9026
Anticoagulant therapy (rivaroxaban vs. placebo)	0.800 (0.327–1.956)	0.6244	0.863 (0.338–2.201)	0.7574

Abbreviations: ACS, acute coronary syndrome; BMS, bare-metal stent; CI, confidence interval; DES, drug-eluting stent; HR, hazard ratio; MI, myocardial infarction; NSTEMI, non-ST segment elevation myocardial infarction; ST, stent thrombosis; STEMI, ST segment elevation myocardial infarction.

Supplementary Material

Figure S1. Cumulative incidence of stent thrombosis stratified by age category (≥ 75 years vs. < 75 years): (A) standard Kaplan-Meier method, (B) landmark analysis

(A)

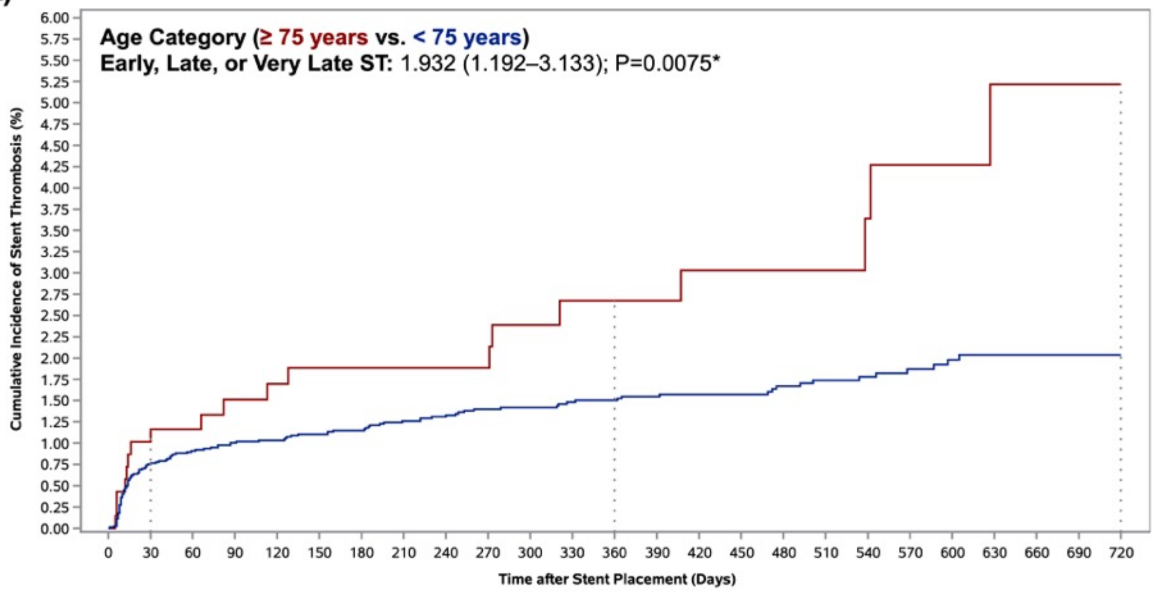

(B)

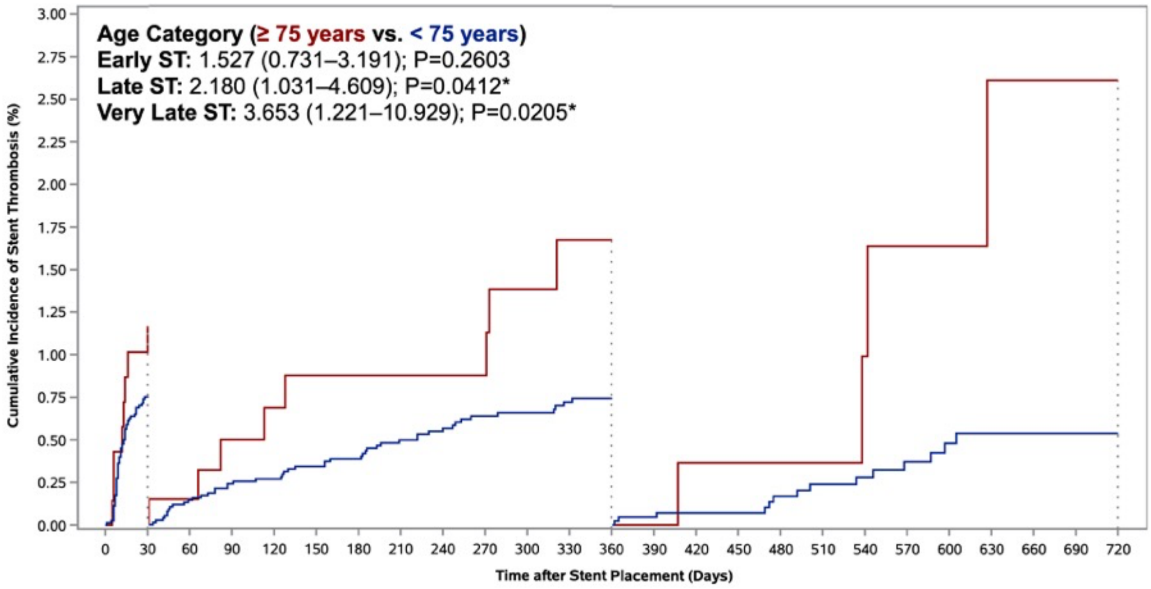

Supplementary Material

Figure S2. Cumulative incidence of stent thrombosis stratified by prior myocardial infarction: (A) standard Kaplan-Meier method, (B) landmark analysis

(A)

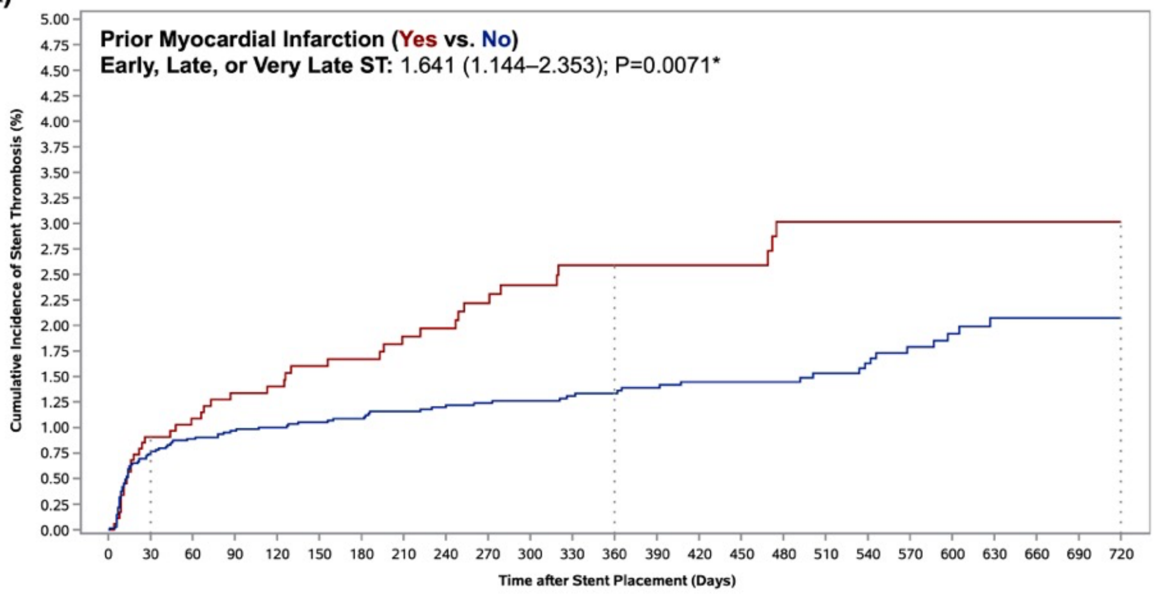

(B)

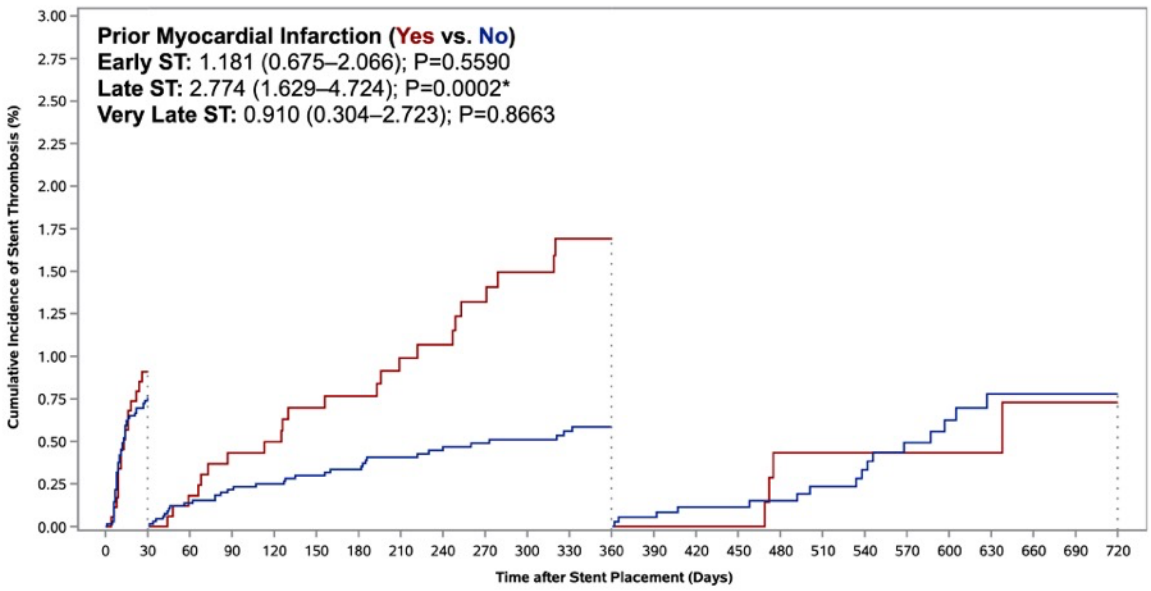

Supplementary Material

Figure S3. Cumulative incidence of stent thrombosis stratified by hemoglobin category (low vs. normal): (A) standard Kaplan-Meier method, (B) landmark analysis

(A)

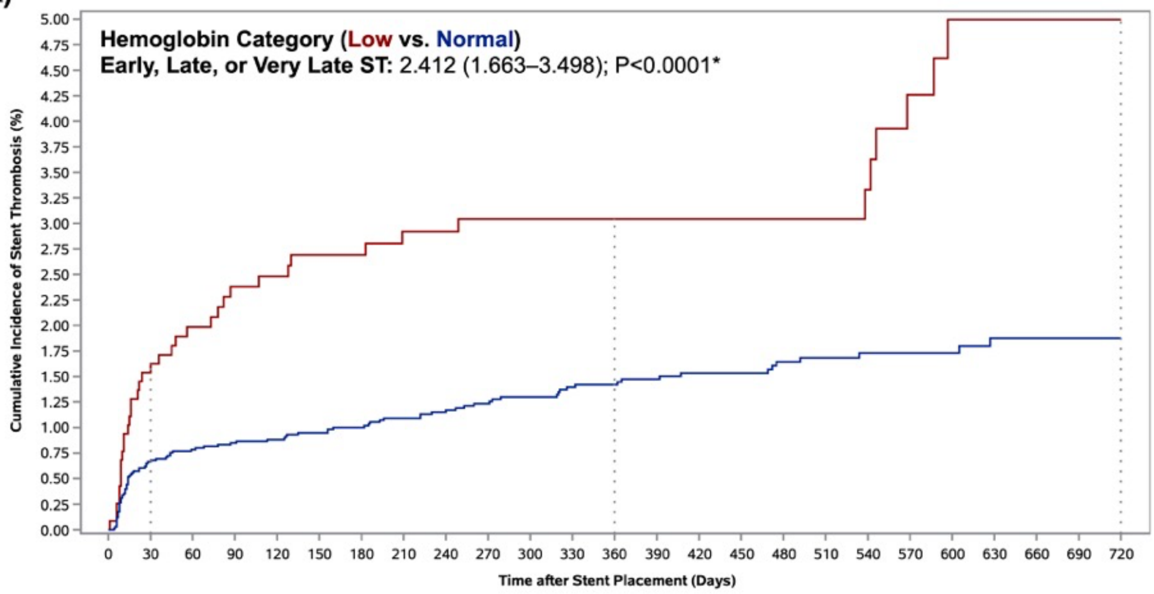

(B)

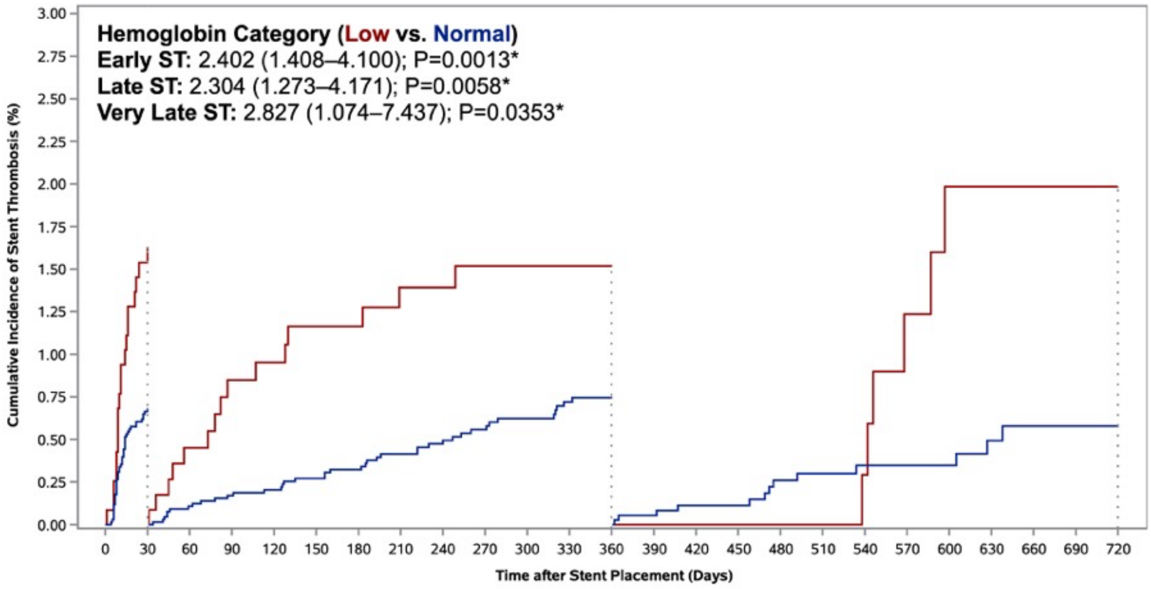

Supplementary Material

Figure S4. Cumulative incidence of stent thrombosis stratified by white blood cell category (high vs. normal): (A) standard Kaplan-Meier method, (B) landmark analysis

(A)

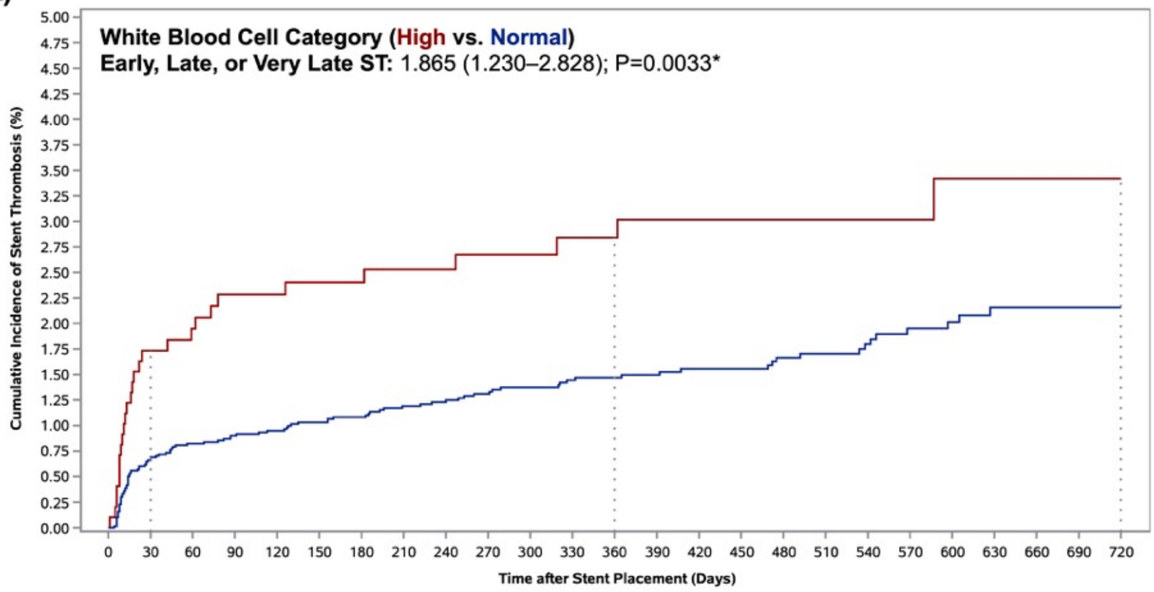

(B)

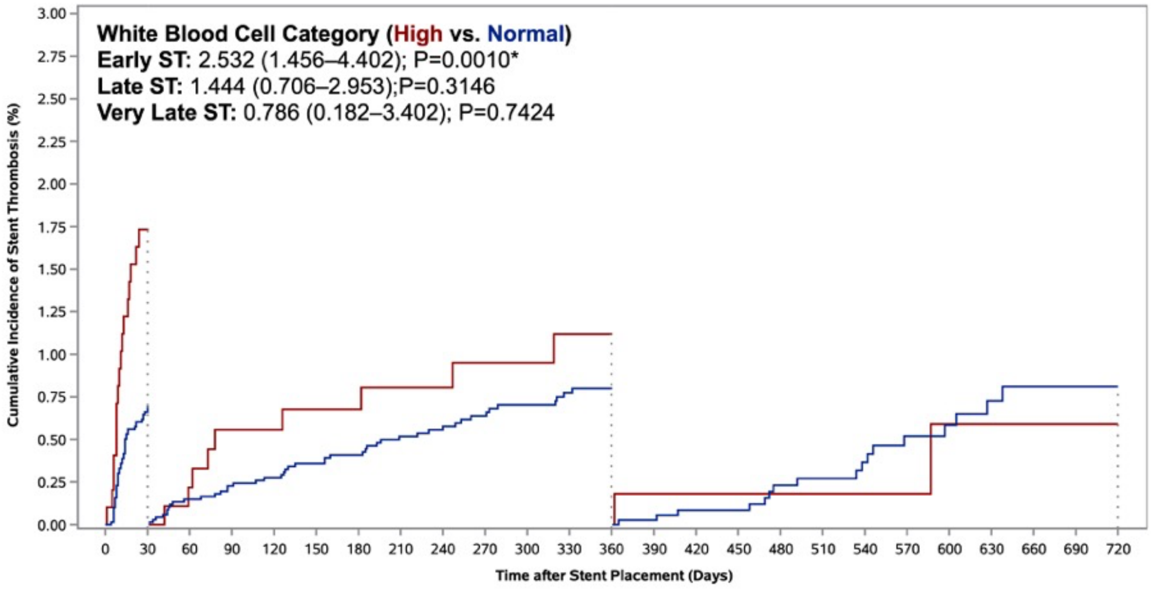

Supplementary Material

Figure S5. Cumulative incidence of stent thrombosis stratified by stent type (drug-eluting stent vs. bare-metal stent): (A) standard Kaplan-Meier method, (B) landmark analysis

(A)

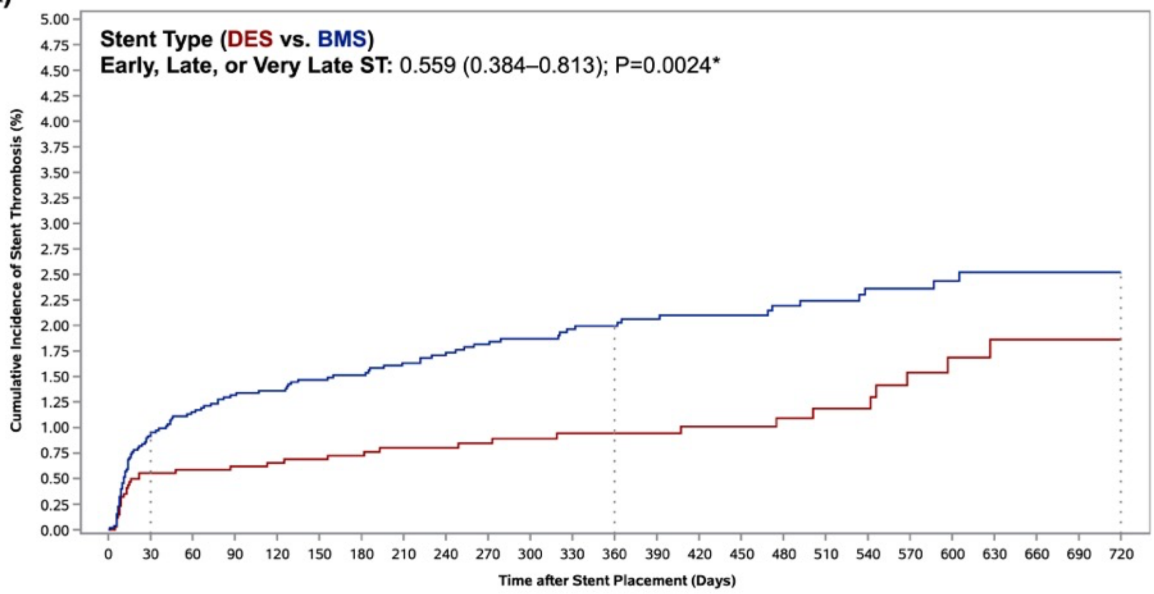

(B)

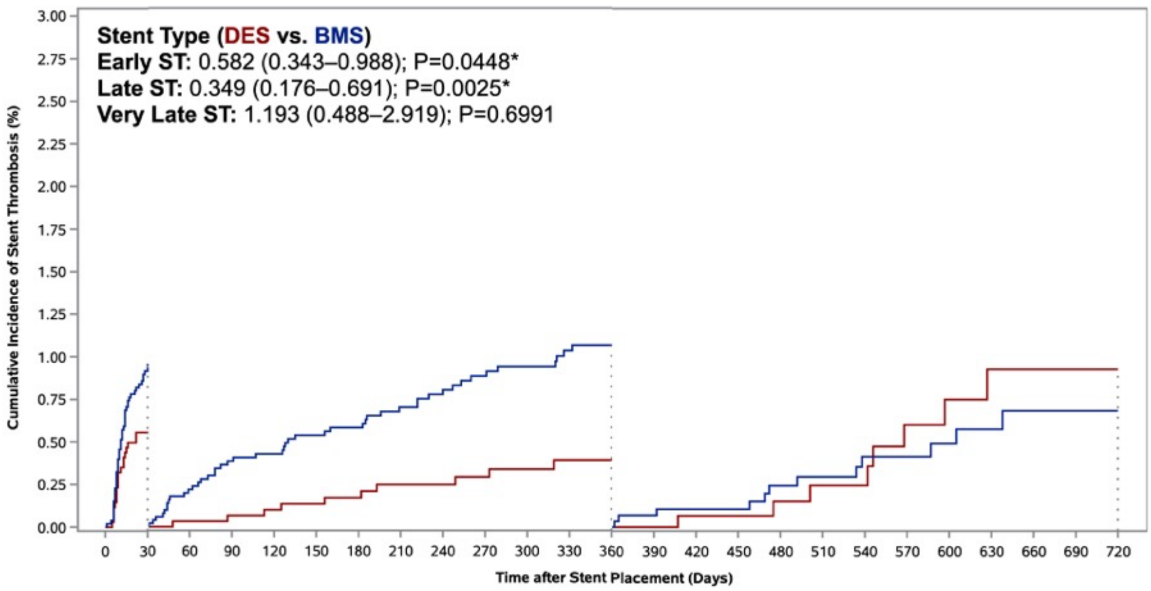

Supplementary Material

Figure S6. Cumulative incidence of stent thrombosis stratified by anticoagulant therapy (rivaroxaban vs. placebo): (A) standard Kaplan-Meier method, (B) landmark analysis

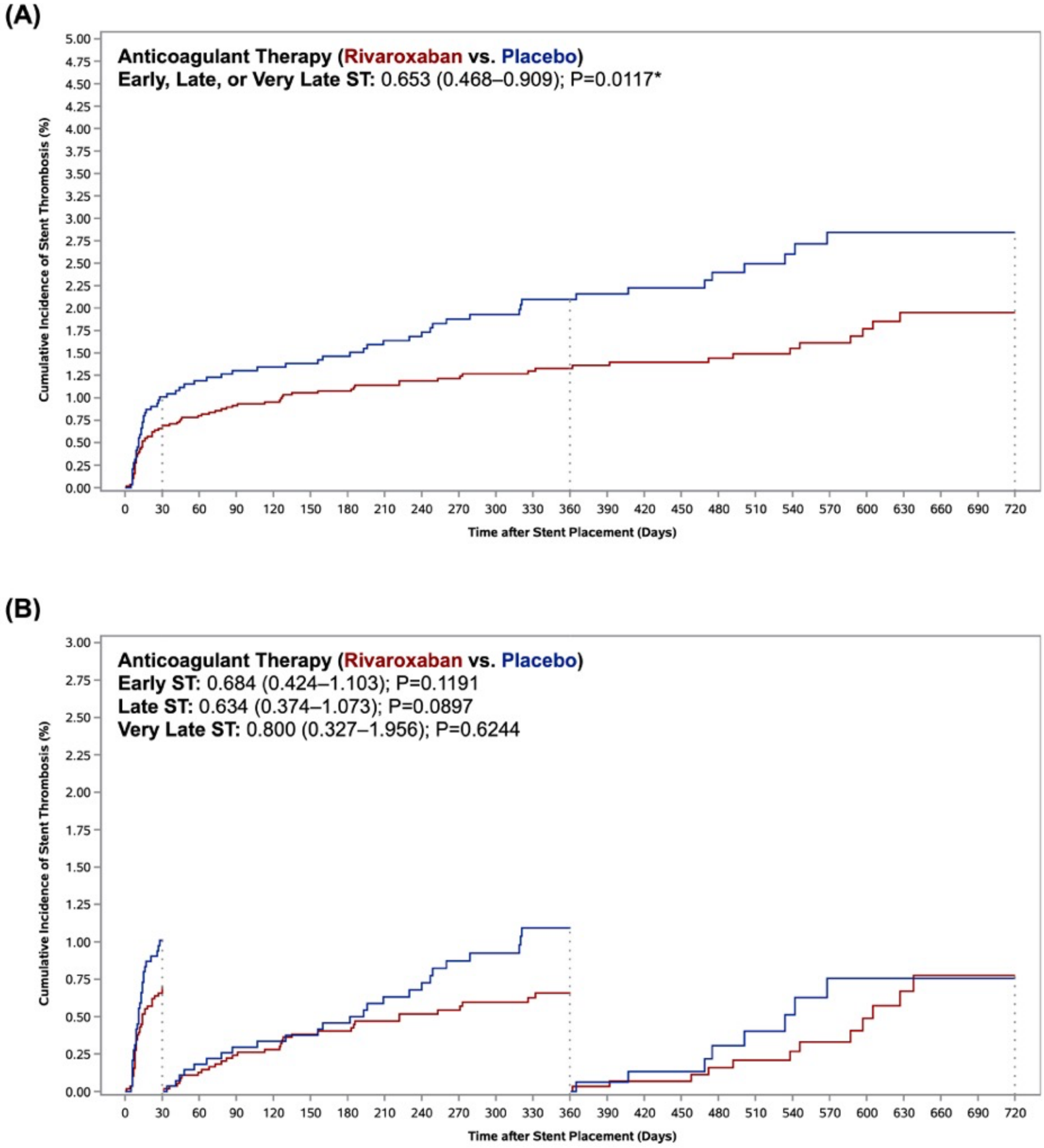

Supplement: Supplementary file 1 [file Presentation1.pdf]
